# Cytoprotective effects of urinary trypsin inhibitor on astrocytes injured by sustained compression

**DOI:** 10.1007/s11033-013-2976-6

**Published:** 2014-01-03

**Authors:** Shuang Zhang, Rongguo Yu, Yingrui Zhang, Kai Chen

**Affiliations:** SICU, Fujian Provincial Hospital, Fujian Medical University Affiliated Provincial Teaching Hospital, Fuzhou, 350001 China

**Keywords:** Astrocytes, Compression injury, LDH release, UTI, Cytoprotection

## Abstract

Decreased cell membrane integrity is a primary pathological change observed in traumatic brain injury (TBI) that activates a number of complex intercellular and intracellular pathological events, leading to further neural injury. In this paper, we assessed the effects of urinary trypsin inhibitor (UTI) on astrocyte membrane integrity by determining the percentage of lactate dehydrogenase (LDH) released after sustained compression injury using a hydrostatic pressure model of mechanical-like TBI. Astrocytes isolated from SD rat pups were injured by sustained compression. At a pressure of 0.3 MPa for 5 min, a significant increase in LDH release was observed compared with control samples. Astrocytes displayed extensive structural disruption of mitochondrial cristae reflected in their swelling. Based on our initial results, injured astrocytes were treated with UTI at a final concentration of 500, 1,000, 3,000 or 5,000 U/ml for 24 h. The percentage of LDH released from injured astrocytes was significantly decreased when 1,000 and 3,000 U/ml of UTI were used. In a separate experiment, astrocytes were treated with UTI at a final concentration of 1,000 U/ml immediately, or at 30 min, 2, 6, or 24 h after sustained compression. The percentage of LDH release was significantly reduced (*P* < 0.05) when astrocytes were treated with UTI immediately or 30 min later. Together, our results suggest that UTI may have protective effects on astrocytes injured by sustained compression injury. Furthermore, the early administration (<2 h after injury) of UTI may result in a better outcome compared with delayed administration.

## Introduction

Traumatic brain injury (TBI) is a significant cause of disability and death worldwide, and its incidence is rising sharply. Current medical therapies exhibit limited efficacy in reversing the initial damage and patients’ prognosis remains poor [1], creating a significant burden for individuals, families and the society in general.

Early research on TBI focused almost exclusively on the injury to neurons with little attention paid to astrocytes. However, in the past decade, many new discoveries led to the recognition of a critical role for astrocytes in the outcome of TBI. Astrocytes, a sub-type of glial cells, are ubiquitous throughout all regions of the central nervous system (CNS). They outnumber neurons by more than fivefold and contiguously tile the entire CNS [1]. Normal astrocytes perform several functions, including: metabolic support, regulation of blood flow, formation of the blood brain barrier (BBB), transmitter uptake and release, modulation of synaptic transmission, and regulation of breathing [2–8]. Astrocytes are vulnerable to CNS insults and are severely damaged and die following TBI [9, 10]. Subsequently, reactive astrogliosis occurs in surviving astrocytes. Reactive astrogliosis involves changes in gene expression, morphology and proliferation in a gradated fashion in relation to the severity of the injury [11–13]. Although the exact details of the role of reactive astrocytes are not completely understood, transgenic mice experiments revealed that ablation of reactive astrocytes after CNS injury causes substantial neuronal inflammation, degeneration, and death [14–16]. Therefore, astrocyte function is crucial for neurons, and affects outcomes following TBI, making astrocytes an important therapeutic target for neuronal protection following brain injury [17].

Damage to the integrity of cell membranes is the primary pathological change observed in mechanically injured cells [18]. It is a major contributor to the development of neuronal damage because it results in the increased accumulation of intracellular sodium, water and calcium ions, in leakage of enzymes and cofactors, and in the activation of cellular pathways [18]. Therefore we tested a therapeutic strategy targeting the protection of damaged cell membranes following TBI. Urinary trypsin inhibitor (UTI), one of the serine-type protease inhibitors isolated from human urine, inhibits not only a variety of proteases such as trypsin, α-chymotrypsin, leukocyte elastases, and cathepsin G, but also hyaluronidase [19]. Several studies demonstrated that UTI may protect against systemic inflammation and reduce the injury induced by pancreatitis, sepsis and ischemia–reperfusion [20–22]. In CNS diseases, UTI may have therapeutic effects in craniocerebral injuries, since it decreases post-ischemic brain edema, oxidative stress, early inflammation and ischemia-reperfusion injury [23, 24]. However, UTI’s protective effects against astrocyte membrane damage after TBI have not been reported. Our study demonstrates that UTI may have a cytoprotective effect on astrocytes injured by sustained compression injury.

## Materials and methods

### Ethics statement

All procedures followed the National Guide for the Care and Use of Laboratory Animals. All animal experiments were approved by the Fujian Provincial Hospital Committee on Ethics in the Care and Use of Laboratory Animals.

### Cell culture

Astrocyte cultures were prepared using a previously described method [25, 26]. Cortices were isolated from 1- to 2-day-old rat pups, cleaned of white matter and meninges, minced, and digested with 0.25 % trypsin–EDTA (GIBCO, Invitrogen Inc., Carlsbad, CA, USA) for 20 min in a 37 °C water bath. Cells were next diluted into Dulbecco’s modified Eagle medium/nutrient mixture F-12 (DMEM/F12, Hyclone, Thermo Fisher Scientific Inc., Waltham, MA, USA) supplemented with 10 % fetal bovine serum (FBS, Hyclone, Thermo Fisher Scientific Inc., Waltham, MA, USA), washed, triturated, counted and seeded into 25-cm^2^ flasks (NUNC, Thermo Fisher Scientific Inc., Waltham, MA, USA) at an initial density of 0.5–1 × 10^6^ cells per flask. Flasks were cultured at 37 °C in 5 % CO_2_. The medium was changed every 2–3 days until culture attained confluence. To remove neurons, microglia and oligodendrocyte precursor cells, flasks were shaken at 250 rpm for 18 h at 37 °C in a temperature-controlled orbital shaker (Shanghai Fuma Laboratory Equipment Co., Ltd., Shanghai, China). After medium removal, cells were trypsinized for 5-7 min. The trypsin was diluted with DMEM/F12 supplemented with 10 % FBS, thus allowing the cells to lift from the flask bottom. The lifted cells were washed, centrifuged, counted, and seeded into 25-cm^2^ flasks at a density of 0.5–1 × 10^6^ cells per flask. Astrocyte cultures were used in experiments for a total of 4 weeks after removal from rats. Cultures consisted of at least 95 % astrocytes as determined by glial fibrillary acidic protein (GFAP) immunocytochemistry.

### Cell injury

Astrocytes for injury experiments were placed in 48-well (1.1 cm^2^ each) multidishes (NUNC, Thermo Fisher Scientific Inc., Waltham, MA, USA). When the cells reached a confluent monolayer, the DMEM/F12 was changed to serum-free medium 1 day before the experiment. Astrocytes were injured using a pressure device developed by Murphy and Horrocks [27]. Pressure was induced by increasing atmospheric pressure within a stainless steel chamber (19.5 cm in length, 19.5 cm in width, and 20 cm in height). The chamber contained high-purity compressed air (78 % nitrogen, 21 % oxygen, 0.93 % argon, 0.04 % carbon dioxide). Pressure duration time was 1 or 5 min with a pressure ranging from 0.1 to 0.3 MPa. The optimal duration time and pressure were determined based on the percentage of lactate dehydrogenase (LDH) released. Control cultures were incubated at 37 °C in 5 % CO_2_ during experimentation.

### UTI administration

Astrocytes for experiments were placed in 48-well (1.1 cm^2^ each) multidishes. When the cells reached a confluent monolayer, the incubated medium for cells was changed to serum-free medium 1 day before the experiment. 0.3 MPa of pressure was administered to astrocytes for 5 min. Subsequently, the injured cells were treated with UTI (Techpool Bio-Pharma Co. Ltd., Guangzhou, China), dissolved in serum-free culture medium at final concentrations of 100, 1,000, 3,000 and 5,000 U/ml, and maintained for 24 h.

Based on the results of the UTI intervention experiment, astrocytes plated in 48-well multidishes were randomly divided into 5 groups, according to the time of UTI exposure; each group comprised 3 subgroups: control group, injury group, and UTI intervention group. Cells in the UTI group were treated with UTI at a final concentration of 1,000 U/ml immediately, or at 30 min, 2 h, 6 h, and 24 h after sustained compression (0.3 MPa, 5 min).

### LDH release

The degree of damage to cell membrane integrity has previously been evaluated by measuring the percentage of intracellular LDH released [18, 27–29]. LDH was measured using an in vitro toxicology assay kit (Sigma, St. Louis, MO, USA). The assay is based on the reduction reaction of nicotinamide adenine dinucleotide (NAD) by LDH [30]. The extracellular medium was sampled after the mechanical stimulus. The remaining intracellular LDH was also measured after solubilizing the cells with 0.2 % Triton X-100. The LDH release rate (%) was calculated using the following equation:

$${\text{LDH}}\,{\text{release}}\,(\% ) = \frac{{{\text{Extracellurar}}\,{\text{LDH}}\, \times \,100}}{{{\text{Intracellular}}\,{\text{LDH}}\, + \,{\text{Extracellular}}\,{\text{LDH}}}}$$

### Transmission electron microscopic examination

Transmission electron microscope (TEM) was used to examine the ultrastructural details of injured astrocytes. Astrocytes were plated in 6-well (9.6 cm^2^ each well) multidishes (NUNC, Thermo Fisher Scientific Inc., Waltham, MA, USA). First, 0.3 MPa of pressure was administered to a monolayer of astrocytes for 5 min. Next, each well was rinsed with phosphate-buffered saline (PBS, pH 7.4, GIBCO, Life Technologies Co., Waltham, MA, USA), and trypsinized for 4–5 min. The trypsinized cells were diluted with DMEM/F12 supplemented with 10 % FBS. The lifted cells were centrifuged and fixed with 3 % glutaraldehyde and 1.5 % paraformaldehyde in PBS at room temperature for 4 h. After being rinsed with PBS, samples were fixed with 1 % osmic acid and 1.5 % potassium ferrocyanide at 4 °C for 1.5 h. Samples were then dehydrated with graded ethanol and acetone, embedded in epoxy resin, and cut into ultra-thin slices of about 70–80 nm. Slices were stained with uranyl acetate and lead citrate, and observed under an EM208 TEM (Philips, Royal Dutch Philips Electronics Ltd., Best, The Netherlands).

### Statistical analysis

All data are presented as mean ± standard deviation (SD). Statistical analyses were performed using the SPSS 13.0 software (SPSS Inc., Chicago, IL, USA). Statistical significance was established by one-way analysis of variance (ANOVA) followed by least significant difference (LSD) *t* tests. *P* values <0.05 were considered to be significant.

## Results

### Pressure-induced cell LDH release and morphological changes

**Fig. 1:**
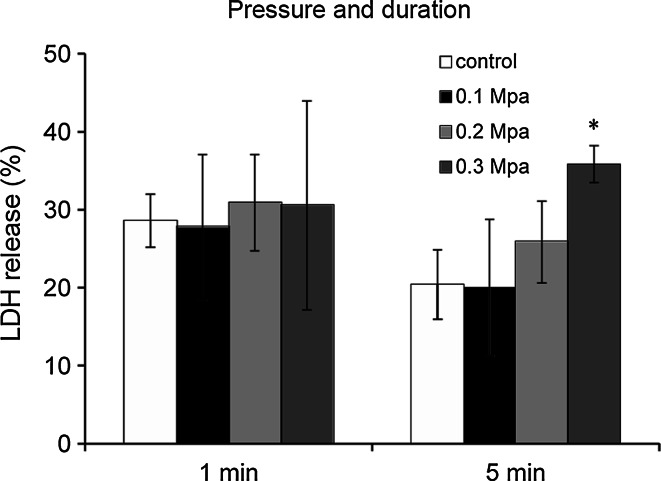
Effects of sustained compression on LDH release from astrocytes. Pressure duration of 1 or 5 min was used on astrocytes over a pressure of 0.1, 0.2, or 0.3 MPa. LDH release was expressed as a percent of the total releasable LDH (extracellular and intracellular LDH). Values are presented as mean ± SD. *P < 0.01 versus control

**Fig. 2:**
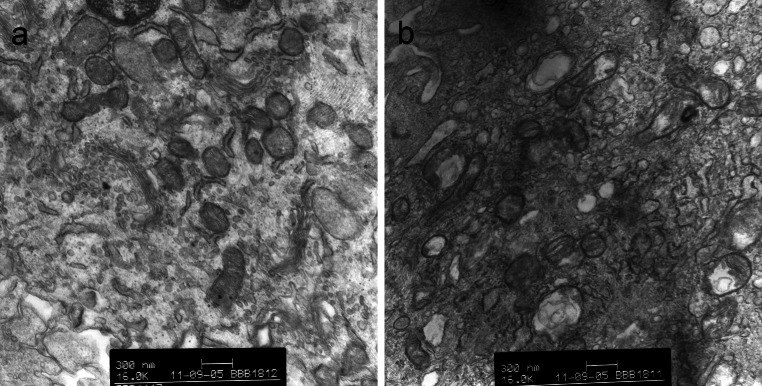
Astrocyte morphology before and after sustained compression injury. **a** Normal ultrastructural features of uninjured astrocytes. **b** Ultrastructural abnormalities in astrocytes injured by sustained compression injury (0.3 MPa for 5 min)

No significant increase in the percentage of LDH release was detected after exposure to increased pressure for a short duration of time (1 min). When the duration of exposure was increased to 5 min, a significant increase in LDH release from astrocytes was observed at a pressure of 0.3 MPa (Fig. 1). Astrocyte morphology was studied using TEM. Uninjured astrocytes in the control group showed normal ultrastructural features (Fig. 2a). On the other hand, injured astrocytes displayed structural abnormalities, including endoplasmic reticulum swelling, mitochondrial cristae disruption and Golgi apparatus vacuolization (Fig. 2b). Therefore, in the following experiments, a pressure of 0.3 MPa for 5 min was used to establish the sustained compression trauma model of astrocytes.

### Protective effect of UTI on injured astrocytes

**Fig. 3:**
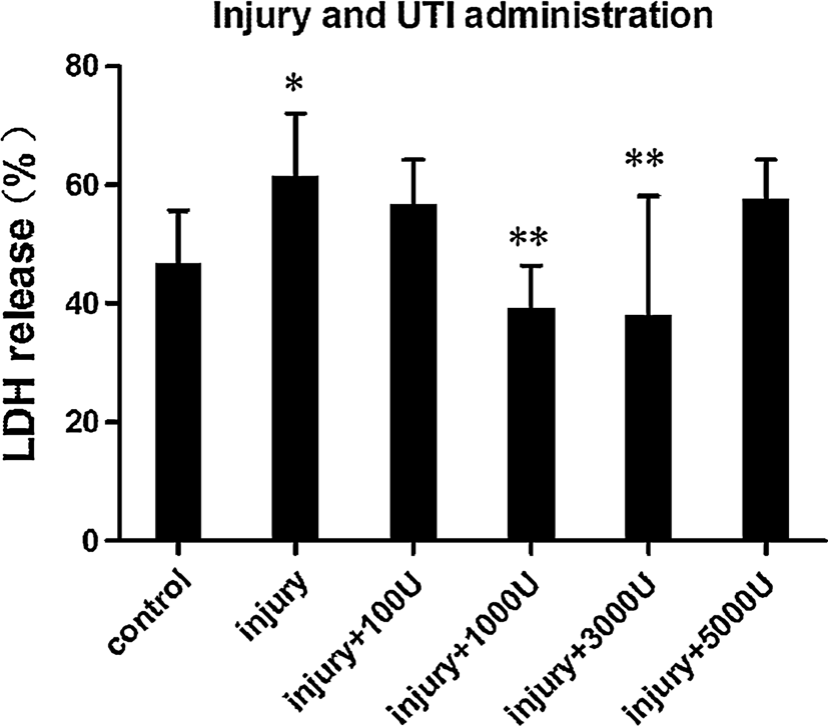
Effects of different concentrations of UTI on LDH release from injured astrocytes. *P < 0.05 versus control; **P < 0.01 versus injury. The amount and duration of pressure for the injury and UTI group were 0.3 MPa and 5 min. LDH release was expressed as a percent of the total releasable LDH (extracellular and intracellular LDH). Values are presented as mean ± SD

The percentage of LDH release increased significantly in injury samples (0.3 MPa, 5 min) compared with the control samples. This result demonstrated that the astrocytes were injured by sustained compression. After being treated with UTI at concentrations of 1,000 and 3,000 U/ml for 24 h, astrocytes released significantly less LDH compared with injury samples. However, a higher UTI concentration (5,000 U/ml) had no additional protective effect (Fig. 3).

**Fig. 4:**
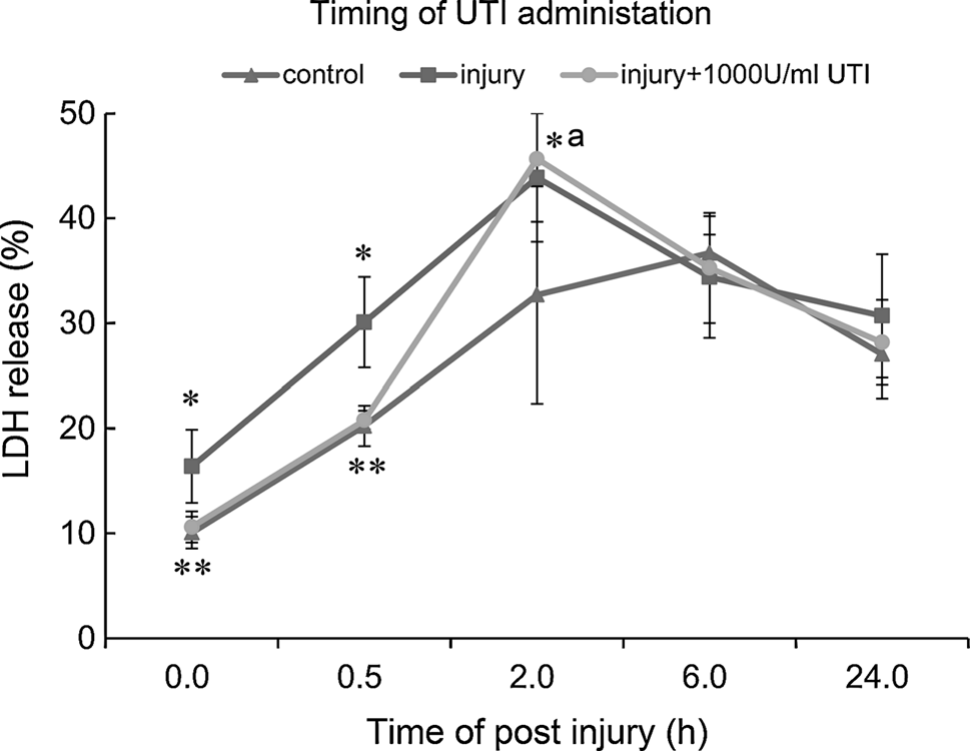
Time-dependent effects of UTI on LDH release from injured astrocytes. *P < 0.01 versus control; *^a^ *P* < 0.05 versus control; **P < 0.01 versus injury. The amount and duration of pressure for the injury and UTI groups were 0.3 MPa and 5 min. LDH release was expressed as a percent of the total releasable LDH (extracellular and intracellular LDH). Values are presented as mean ± SD

In addition, the time-dependent effect of UTI administration on LDH release after injury was also studied. After being treated with 1,000 U/ml of UTI immediately, or 30 min post injury, astrocytes released significantly less LDH compared with untreated injury samples. The protective effect was lost when astrocytes were treated with UTI 2 h post-injury. At later time points, the percentage of LDH released from injured astrocytes declined and no statistically significant differences in LDH release were detected among control, injury and UTI-treated group at 6 and 24 h post-injury (Fig. 4). This result demonstrated that UTI may have protective effects on astrocytes at the early stage of injury (<2 h after injury), and that membrane damage may be irreversible following an early window of recovery. Finally, our data also suggest that based on LDH release, astrocytes eventually recovered from pressure-induced injury in our model and that this recovery was unaffected by UTI.

## Discussion

Using a hydrostatic pressure model of mechanical-like TBI, we studied the presumptive cytoprotective effects of UTI on injured astrocytes in vitro. Although in vitro brain injury models cannot surpass in vivo models, the former are able to complement the latter, explore different aspects, provide better control over experimental variables, and fulfill real time and spatial measurement of biological and mechanical parameters [31, 32]. The model employed in our research was a nonelastic chamber where a constant hydrostatic pressure was applied for a defined time, and compressed air was used as the gas source in order to eliminate potentially confounding factors such as ischemia and hypoxia. In cells subjected to a high-pressure environment, once the lateral compressions exceed the tolerance of cell membranes, cellular membrane integrity is compromised. According to the studies of Murphy and Horrocks [27], the effect of sustained hydrostatic pressure-induced injury depended on pressure and pressure duration, and these two parameters varied for different types of cells. Based on these results, we tested pressure durations of 1 and 5 min over a pressure range of 0.1, 0.2, and 0.3 MPa on astrocytes. At a pressure of 0.3 MPa during 5 min, a significant increase in LDH released from astrocytes was observed. The increase in cytoplasmic enzyme efflux is an indicator of compromised cell membrane integrity. In addition, injured astrocytes displayed ultrastructural abnormalities, including endoplasmic reticulum swelling, mitochondrial cristae disruption and Golgi apparatus vacuolization, and these abnormalities were in accordance with an increased LDH release. These morphological abnormalities implied that certain cell functions may be impaired, such as the production and transport of proteins, energy conversion, and oxidative phosphorylation. These results show that a hydrostatic pressure model of mechanical-like TBI on astrocytes was successfully established, and that the injured cells were characterized by decreased cell membrane integrity and ultrastructural abnormalities. Thus, LDH assay alone was used in our experiments.

Cell membrane integrity damage is a primary pathological change after mechanical neuronal injury. It is also the major contributor to the development of neuronal damage resulting in ionic imbalances and activation of several cellular pathways [18]. There are several ways to assess cell membrane integrity, including vital dye staining and measurement of cytoplasmic enzyme release. Vital dyes, such as trypan blue and propidium iodide (PI), are easily used and cheap, but they are usually time-consuming and do not allow the processing of a large number of samples. Furthermore, they do not account for the proportion of dead cells, and the actual rate of cell death may thus be underestimated [33]. In contrast, measurement of cytoplasm enzymes release is more accurate, easily automated, and reliably quantifies neuroprotective effects [34], and LDH is the most commonly assessed among these enzymes. Compared with other released enzymes (such as glucose-6-phosphate dehydrogenase, which has a half-life of ~2 h in culture medium), LDH is more stable [35]. Moreover, the percentage of LDH released was used to characterize membrane damage instead of the amount of LDH released. Apart from cell membrane integrity, the amount of LDH efflux is also associated with the number of cells present. Therefore, the percentage of LDH release, a ratio of the amount of LDH efflux to the total LDH, was used to eliminate the influence of the number of cells on our quantification of LDH release.

Our studies demonstrated that the treatment of injured astrocytes with UTI at a concentration of 1,000 or 3,000 U/ml significantly improved cell membrane integrity. In addition, the timing of UTI administration was also critical. The mechanism underlying the cytoprotective effects of UTI on injured astrocytes may involve the inhibition of hydrolytic enzymes released from lysosomes. Lysosomes are membrane-bound vesicles serving as the cell’s main digestive compartment. Lysosomes contain numerous hydrolytic enzymes, including proteases, peptidases, phosphatases, nucleases, sulphatases and lipases. Together, these hydrolytic enzymes are capable of digesting all cellular components. In healthy cells, the lysosomal membrane protects cellular components from these degradative enzymes [36]. However, acute brain injury such as trauma, seizures and ischemia can result in the increase of free intracellular calcium and zinc. Calcium and zinc toxicity may contribute to lysosomal membrane permeabilization, disintegration and leakage of hydrolytic enzymes leading to the digestion of cellular components. This process is one of the major cascades involved in astrocyte necrosis [37–41]. Lysosomal enzyme inhibitors may therefore be neuroprotective in brain injury. Being a serine-type protease inhibitor, UTI may protect cell proteins from digestion by inhibiting lysosomal proteases. In this way, UTI may stabilize the membrane of lysosomes and promote the resealing of disrupted cell membranes. However, increasing UTI dose to 5,000 U/ml had no further effect compared with the 3,000 U/ml dose. This may be due to the fact that UTI is an inhibitor of a variety of proteases and that high UTI doses may interfere with normal cellular proteolytic processes, thus counter-balancing its beneficial effects.

In conclusion, UTI may have cytoprotective effects on astrocytes injured by sustained compression trauma due to the improvement of cell membrane integrity. Time-dependent treatment is essential as the early administration (<2 h after injury) of UTI resulted in better protection compared with the delayed administration.
